# High-Performance Deep Neural Network-Based Tomato Plant Diseases and Pests Diagnosis System With Refinement Filter Bank

**DOI:** 10.3389/fpls.2018.01162

**Published:** 2018-08-29

**Authors:** Alvaro F. Fuentes, Sook Yoon, Jaesu Lee, Dong Sun Park

**Affiliations:** ^1^Department of Electronics Engineering, Chonbuk National University, Jeonju, South Korea; ^2^Department of Computer Engineering, Mokpo National University, Muan, South Korea; ^3^Department of Agricultural Engineering, National Institute of Agricultural Sciences (RDA), Jeonju, South Korea; ^4^College of Computer Science and Information Engineering, Tianjin University of Science and Technology, Tianjin, China; ^5^Division of Electronics and Information Engineering, Chonbuk National University, Jeonju, South Korea

**Keywords:** plant diseases, detection, deep neural networks, filter banks, false positives

## Abstract

A fundamental problem that confronts deep neural networks is the requirement of a large amount of data for a system to be efficient in complex applications. Promising results of this problem are made possible through the use of techniques such as data augmentation or transfer learning of pre-trained models in large datasets. But the problem still persists when the application provides limited or unbalanced data. In addition, the number of false positives resulting from training a deep model significantly cause a negative impact on the performance of the system. This study aims to address the problem of false positives and class unbalance by implementing a Refinement Filter Bank framework for Tomato Plant Diseases and Pests Recognition. The system consists of three main units: First, a Primary Diagnosis Unit (Bounding Box Generator) generates the bounding boxes that contain the location of the infected area and class. The promising boxes belonging to each class are then used as input to a Secondary Diagnosis Unit (CNN Filter Bank) for verification. In this second unit, misclassified samples are filtered through the training of independent CNN classifiers for each class. The result of the CNN Filter Bank is a decision of whether a target belongs to the category as it was detected (True) or not (False) otherwise. Finally, an integration unit combines the information from the primary and secondary units while keeping the True Positive samples and eliminating the False Positives that were misclassified in the first unit. By this implementation, the proposed approach is able to obtain a recognition rate of approximately 96%, which represents an improvement of 13% compared to our previous work in the complex task of tomato diseases and pest recognition. Furthermore, our system is able to deal with the false positives generated by the bounding box generator, and class unbalances that appear especially on datasets with limited data.

## Introduction

Plant diseases cause major production and economic loses in the agriculture area. It is nowadays considered as a big issue in the modern agricultural production. Plant protection, in particular, the protection of crops against diseases, has a special role in achieving a higher demand for food and are directly related to the human well-being. Along with the worldwide population, the availability per capita of food is expected to be increased for the next years (Pinstrup-Andersen, 2002). The demand for food is influenced by factors such as the population growth, income levels, urbanization, lifestyles, and preferences (Savary et al., 2012). Therefore, the importance of a proper control during the production process has played an important role in recent times.

An accurate estimation of diseases and pest in plants remains a challenge in the scientific community (Donatelli et al., 2017). Diseases and pest in plants can be generated by several causes (Fuentes et al., 2016) and show different variations throughout their infection status (Fuentes et al., 2017a). Bacteria, fungus, viruses, and insects may result in plant disease and damage (Sankaran et al., 2010). Once infected, a plant develops several symptoms that, if spread, can cause a significant impact on the entire crop. Traditional methods to treat diseases in plants include the use of pesticides. However, an excessive use of pesticides not only increases the cost of production but can also cause an impact on the quality of food. Consequently, a precise estimation of disease incidence, disease severity, and the negative effects of diseases on the quality and quantity of agriculture are important for crop field, horticulture, plant breeding, and improving fungicide efficacy, as well as for plant research (Mahlein, 2016). Monitoring of the growing conditions and detecting diseases in plants is, therefore, critical for sustainable agriculture. In some way, an early detection of suspicious areas in the plant may prevent several economic loses and facilitate the control through appropriate management strategies to increase productivity (Johannes et al., 2017).

Recent interest in neural networks for several areas, and especially their potential applications in agriculture, has fueled the growth of efficient autonomous systems and their application to real problems. Such applications strongly motivate our research in the recognition of pathologies that affect plants, and particularly tomato plants, and at the same time provide a strategy to develop better recognition techniques.

Our previous work (Fuentes et al., 2017b) introduced a detector based on Deep Learning for Tomato Diseases and Pest Recognition, which simultaneously performs the localization and diagnosis of nine different types of diseases and pests. In comparison with other techniques, our system shows the following advantages: (1) It uses images taken in the real field, therefore, we avoid the process of collecting samples and analyzing them in the laboratory; (2) It considers the possibility that a plant can be affected simultaneously by several pathologies in the same sample; (3) It uses images captured by different camera devices with various resolutions; (4) It can efficiently deal with different illumination conditions, size of objects, and background variations, etc.; (5) It provides a practical application in real time that can be used in the field without using expensive and complex technology.

Although the task has been effectively achieved with satisfactory results. We believe that there is still a room that needs to be addressed for this practical application. In fact, we consider that this task remains challenging due to the following conditions: (1) The limited training data with significant unbalanced distribution on the annotated data makes the learning process more biased toward classes with more samples and variations (e.g., leaf mold, canker, plague) while resulting in lower performance in scattered annotated classes with fewer samples (e.g., gray mold, low temperature, powdery mildew). We called this issue a “*class unbalance*” problem. (2) The discrepancy between the classes due to the inter- and intra-class variations results in a high number of false positives that, in fact, limits the system to achieve higher accuracy in this complex recognition task. Consequently, when developing an efficient plant diseases recognition system, it is essential to deal with those problems.

Following our previous approach (Fuentes et al., 2017b), the proposed system uses a refinement diagnosis strategy, which addresses the aforementioned problems, while achieving a higher recognition rate. The main contributions of this paper are summarized as follows. (1) We propose a diagnosis system for an effective recognition of diseases and pests of tomato plants. A primary diagnosis unit detects a set of bounding boxes that are likely containing a disease in the image, then a secondary diagnosis unit verifies bounding boxes detected from the primary diagnosis unit using independent CNN classifiers trained with respect to each class and, finally, an integration unit combines the results from the primary and secondary units to effectively recognize 10 different types of diseases and pests of tomato plant. (2) We introduce a strategy for dealing with false positives generated by object detection networks, and class unbalances problems that work especially on datasets with limited data. (3) By implementing this approach, we are able to obtain a recognition rate of approximately 96% which represents an improvement of 13% compared to our previous work (Fuentes et al., 2017b) in the complex task of tomato plant diseases and pest recognition. It is important to emphasize that our work contrasts with other disease classification-based works (Kawasaki et al., 2015; Mohanty et al., 2016; Sladojevic et al., 2016; Amara et al., 2017; Ferentinos, 2018; Liu et al., 2018), in that, it is a detection-based approach that provides the class and location instances of a particular disease in the image. Furthermore, it uses images from the Tomato Diseases and Pest Recognition Dataset (Fuentes et al., 2017b), which are collected in different field scenarios with real conditions (lighting, background, size, etc.) using several camera devices.

The remainder of this paper is organized as follows. A detailed review of works related to our approach is presented in section Related Works. Section Diagnosis System with Refinement Filter Bank introduces the technical details of our diagnosis system. In section Experimental Results, the experimental results show the performance of our system in the task of tomato diseases and pests recognition. Finally, in Section Conclusion and Future Works, we conclude the paper and mention our future works.

## Related works

In this section, we first introduce methods based on neural networks for object detection and recognition. Then, we review some techniques used for detecting anomalies in plants and, finally, investigate advances in false positives reduction.

### Image-based object detection and feature extractors

Recent years have seen an explosion of visual media available through the internet. This large volume of data has brought new opportunities and challenges for neural network applications. Since the first application of Convolutional Neural Networks (CNN) on the image classification task in the ImageNet Large Scale Visual Recognition Competition 2012 (ILSVRC-2012) (Russakovsky et al., 2015) by AlexNet (Krizhevsky et al., 2012), a CNN composed of 8 layers demonstrated an outstanding performance compared to traditional handcrafted-based computer vision algorithms (Russakovsky et al., 2015). Consequently, in the last few years, several deep neural network architectures have been proposed with the goal of improving the accuracy in the same task.

Object detection and recognition have played an important issue in recent years. In the case of detecting particular categories, earlier applications focused on classification from object-centric images (Russakovsky et al., 2012). Where the goal is to classify an image that likely contains an object in it. However, the new dominant paradigm is not only to classify but also precisely localize objects in the image (Szegedy et al., 2013). Consequently, current state-of-the-art object methods for object detection are mainly based on deep CNNs (Russakovsky et al., 2015). They have been categorized into two types: two-stage and one-stage methods. Two-stage methods are commonly related to the Region-based Convolutional Neural Networks, such as Faster R-CNN (Ren et al., 2016), Region-based Fully Connected Network (R-FCN) (Dai et al., 2016). In these frameworks, a Region Proposal Network (RPN) generated a set of candidate object locations in the first stage, and the second stage classifies each candidate location as one of the classes or background using a CNN. It uses a deep network to generate the features that are posteriorly used by the RPN to extract the proposals. In addition to systems based on region proposals, one-stage frameworks have been also proposed for object detection. Most recently SSD (Liu et al., 2016), YOLO (Redmon et al., 2015) and YOLO V2 (Redmon and Farhadi, 2017) have demonstrated promising results, yielding real-time detectors with accuracy similar to two-stage detectors.

Over the last few years, it has been also demonstrated that deeper neural networks have achieved higher performance compared to simple models in the task of image classification (Russakovsky et al., 2015). However, along with the significant performance improvement, the complexity of deep architectures has been also increased, such as VGG (Simonyan and Zissermann, 2014), ResNet (He et al., 2016), GoogLeNet (Szegedy et al., 2015), ResNeXt (Xie et al., 2017), DenseNet (Huang et al., 2017), Dual Path Net (Chen et al., 2017) and SENet (Hu et al., 2017), etc. As a result, deep artificial neural networks often have far more trainable model parameters than the number of samples they are trained on (Zhang et al., 2017). Despite using large datasets, neural networks are prone to overfitting (Pereyra et al., 2017). On the other hand, several strategies have been applied to improve performance in deep neural networks. For example, data augmentation to increase the number of samples (Bloice et al., 2017), weights regularization to reduce model overfitting (Van-Laarhoven, 2017), randomly dropping activations with Dropout (Srivastava et al., 2014), batch normalization (Ioffe and Szegedy, 2015). Although these strategies have proven to be effective in large networks, the lack of data or class unbalances problems for several applications are still a challenge to deal with. There is no a certain way yet of understanding the complexity of artificial neural networks for their application to any problem. Therefore, the importance of developing strategies that are designed specifically for applications that include limited data and class unbalance issues. In addition, depending on the complexity of the application, the challenge nowadays is to design deep learning methods that can perform a complex task while maintaining a lower computational cost.

### Anomaly detection in plants

The problem of plant diseases is an important issue that is directly related to the food safety and well-being of the people. Diseases and pest affect food crops, that in turn causes significant losses in the farmer's economy. The effects of diseases on plants are becoming a challenging approach in terms of crop protection and production of healthy food. Traditional methods for the identification and diagnosis of plant diseases depend mainly on the visual analysis of an expert in the area, or a study in the laboratory. These studies generally require a high professional knowledge in the field, beside the probability of failure to successfully diagnose specific diseases, which consequently led to erroneous conclusions and treatments (Ferentinos, 2018). Under those circumstances, to obtain a fast and accurate decision, an automatic system would offer a highly efficient support to identify diseases and pest of infected plants (Mohanty et al., 2016; Fuentes et al., 2017b). Recent advances in computational technology, in particular, Graphics Processing Units (GPUs), have led to the development of new image-based technology, such as high efficient deep neural networks. The application of deep learning has been also extended to the area of precision agriculture, in that, it has shown a satisfactory performance when dealing with complex problems in real time. Some applications include the study of diseases identification of several crops, such as tomato (Fuentes et al., 2017b), apple (Liu et al., 2018), banana (Amara et al., 2017), wheat (Sankaran et al., 2010), cucumber (Kawasaki et al., 2015).

CNN-based methods constitute a powerful tool that has been used as a feature extractor in several works. Mohanty *et* al. (Mohanty et al., 2016) compare two CNN architectures AlexNet and GoogLeNet to identify 14 crop species and 26 diseases using a large database of diseases and healthy plants. Their results show a system that is able to efficiently classify images that contain a particular disease in a crop using transfer learning. However, the drawback of this work is that its analysis is only based on images that are collected in the laboratory, not in the real field scenario. Therefore, it does not cover all the variations included there. Similarly, Sladojevic et al. (2016) identify 13 types of plant diseases out of healthy leaves with an AlexNet CNN architecture. They used several strategies to avoid overfitting and improve classification accuracy, such as data augmentation techniques to increase the dataset size, and finetuning to increase efficiency while training the CNN. The system achieved an average accuracy of 96.3%. Recently, Liu et al. (2018) proposed an approach for apple leaf disease identification based on a combination of AlexNet and GoogLeNet architectures. Using a dataset of images collected in the laboratory, that system is trained to identify four types of apple leaf diseases with an overall accuracy of 97.62%. In (Ferentinos, 2018), Ferentinos evaluates various CNN models to detect and diagnose plant diseases using leaves images of healthy and infected plants. The system is able to classify 58 distinct plant/disease combinations from 25 different plants. In addition, the experimental results show an interesting comparison when using images collected in the laboratory vs. images collected in the field. Promising results are presented using both types of images, with the best accuracy of 99.53% given by a VGG network. However, the success rate is significantly lower when images collected in the field are used for testing instead of laboratory images. In fact, according to the author, this demonstrates that image classification under real field conditions is much more difficult and complex than using images collected in the laboratory.

Although the works mentioned above show promising results in the task of plant diseases identification, challenges such as the complex field conditions, variation of infection, various pathologies in the same image, surrounding objects, are not investigated. They mainly use images collected in the laboratory, and therefore, do not deal with all the conditions presented in a real scenario. Furthermore, they are diseases classification-based methods.

In contrast, Fuentes et al. (2017b) presented a system that is able to successfully detect and localize 9 types of diseases and pests of tomato plant using images collected in the field, including real cultivation conditions. That approach differs from the others in that it generates a set of bounding boxes that contain the location, size, and class of diseases and/or pest in the image. This work investigates different meta-architectures and CNN feature extractors to recognize and localize the suspicious areas in the image. As a result, the authors show a satisfactory performance of 83%. However, the system presents some difficulties that do not allow it to obtain a higher performance. They mention that due to the lack of samples, some classes with high variability tend to be confused with others, resulting in false positives or lower precision.

Following the idea in (Fuentes et al., 2017b), our current work aims to address the problems mentioned above and improve their results by focusing on false positives and class unbalance issues. On the other hand, our approach studies several techniques to make the system more robust against the inter- and intra-class variations of tomato diseases and pests.

### The problem of false positives

Although the efficiency of object detectors has been improved since deeper neural networks are used as feature extractors, they cannot be generalized for all applications. In addition to the complexity of collecting a dataset for a specific purpose, class unbalance has shown to be a problem when training deep networks for object detection. Consequently, the number of false positives generated by the network is high, which in fact results in a lower precision rate.

In classification problems, the error can be caused by many facts. It can be a measure of true positives (correct classification) and true negatives compared to false positives (false alarms) and false negatives (misses). In object detection, the false positives deserve special attention as they are used to calculate precision. A higher number of false positives yields a lower precision value. Therefore, several techniques have been proposed to overcome this issue. For instance, in (Sun et al., 2016), the problem of object classification and localization is addressed by Cascade Neural Networks that use a multi-stream multi-scale architecture without object-level annotations. In this work, a multi-scale network is trained to propose boxes that likely contain objects, and then a cascade architecture is constructed by zooming onto promising boxes and train new classifiers to verify them. Another approach in (Yang et al., 2016), proposes a technique based on the concept of divide and conquer. Each task is divided via cascade structure for proposal generation and object classification. In proposal generation, they add another CNN classifier to distinguish objects from the background given the output of a previous Region Proposal Network. In the classification task, a binary classifier for each category focuses on false positives caused by mainly inter- and intra-category variances.

### Hard examples mining

In conventional methods, an important assumption to trade off the error generated by the high number of false positives is mentioned in (Viola and Jones, 2001). They suggest that setting a threshold yields classifiers with fewer positives and lower detection rate. Lower thresholds yield classifiers with more false positives and higher detection rate. However, at this point, that concept is unknot yet clear, whether adjusting a threshold preserves the training and helps generalization in deep learning.

Recently, the concept of hard examples mining has been applied to make the training of neural networks easier and efficient. In (Shrivastava et al., 2016), a technique called “Online Hard Example Mining” (OHEM) aims to improve the training of two-stage CNN detectors by constructing mini batches using high-loss examples. This technique removes the need for several heuristic and hyperparameters used in Region-based Convolutional Networks by focusing on the hard-negative examples. In contrast, the scope of this work is to understand whether the use of a refinement strategy can deal with the false positives generated by an object detection network.

The design of our multi-level approach points out two steps for object detection with a specific application in tomato diseases and pest recognition, in particular, the concept of Region-Based Neural Networks for bounding box generation (Fuentes et al., 2017b) and the CNN filter bank for “false positives” reduction. We emphasize that although our previous approach (Fuentes et al., 2017b) shows a satisfactory performance, the results can be further improved with the techniques proposed in our current approach. This aims to make the system more robust to inter- and intra-class variations.

## Diagnosis system with refinement filter bank

### System overview

**Figure 1:**
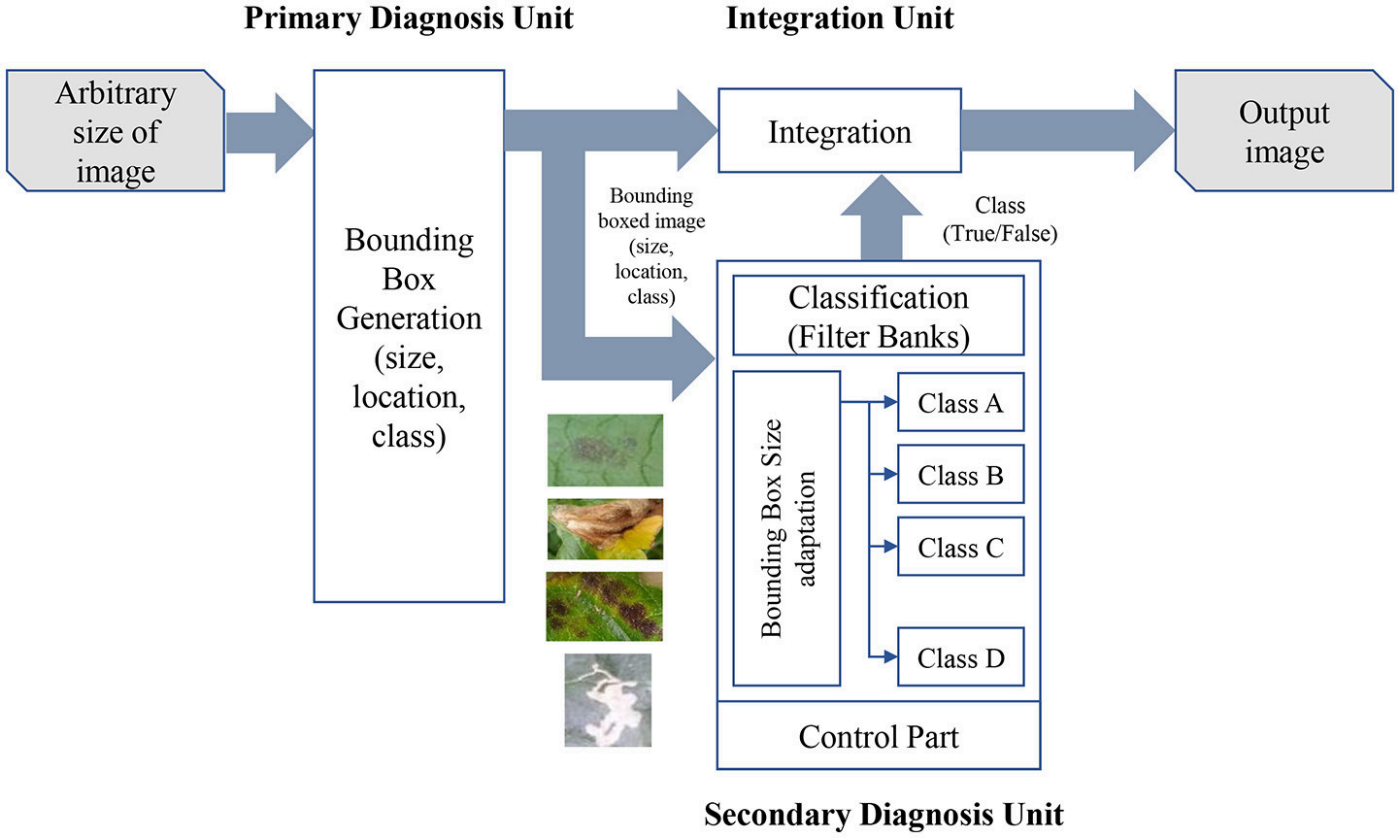
A general overview of our proposed approach. The input images with an arbitrary size are trained in our primary diagnosis unit that generates bounding boxes along with their location and class of the infected areas in the image. The set of bounding boxes is used as input in the secondary diagnosis unit, which independently trains CNN filter banks for each class, with the purpose of reducing the number of false positives generated by the primary unit. Both systems are further integrated into class and location.

Our approach proposes a method to detect diseases and pests of tomato plants using technology based on Deep Learning. The system consists of three basic components: a primary diagnosis unit (Bounding Box Generator), a secondary diagnosis unit (CNN filter bank), and an integration unit. For each image and class category, the primary unit generates a set of bounding boxes with scores of a specific class instance, and the coordinates that indicate the location of the target. Then, the secondary unit filters the confidence of each box by training CNN classifiers independently for each class to further verify their instance. Finally, the integration unit combines the results from the primary and secondary units. Figure 1 illustrates the overall proposed system.

### Primary diagnosis unit

We follow the system proposed in (Fuentes et al., 2017b) that implements a meta-architecture and several feature extractors to handle detection and recognition of complex diseases and pests in images. The input of the system is an image of any arbitrary size. In the first part of the framework, the primary diagnosis unit (bounding box generation) proposes a set of boxes that contain the suspicious areas of the image. That is, for an input image *I* and 10 object categories *C* = {1, 2, 3, …, 10}, we want to extract the object proposals

(1) $$\begin{array}{l} {\text{b}}_{\text{i}}=\left\{{\text{s}}_{\text{i}},\;{\text{l}}_{\text{i}},\;{\text{c}}_{\text{i}}\right\},\;\text{i}=1,2,\ldots ,{\text{B}}_{\text{I}} \end{array}$$

where *B*_*I*_ is the number of bounding boxes detected from the image *I*, and *b*_*i*_ is the *ith* bounding box. The set of bounding boxes provide information such as the size *s*, location *l* and class score *c*.

The following sub-sections show the main characteristics of the primary diagnosis unit.

#### Faster R-CNN for bounding box detection

**Figure 2:**
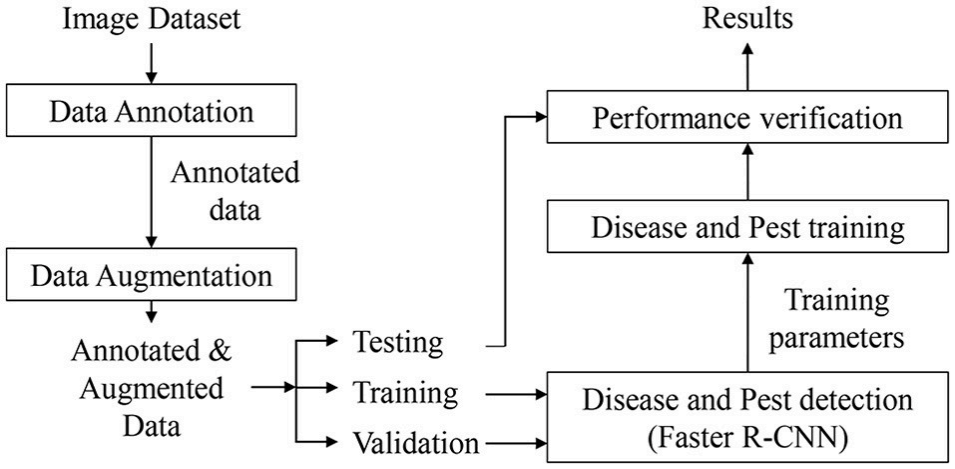
Primary Diagnosis Unit for bounding box detection. Similar to Fuentes et al. (2017b).

**Figure 3:**
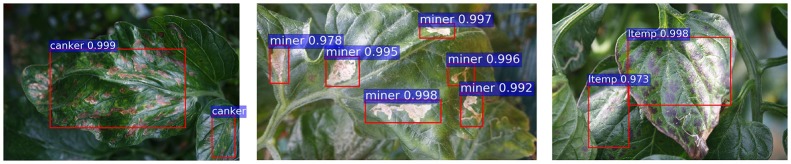
A representation of bounding boxes with various sizes for different detected classes.

Figure 2 shows the process followed by the primary diagnosis unit to detect the suspicious areas containing diseases and pests in the input image. This part is mainly based on the Faster R-CNN. It uses a Region Proposal Network (RPN) to generate a feature map through a CNN and proposes vectors by convolving them using a sliding-window method. The size, location, and class score (probability of having an object or not) are generated for each bounding box proposed by the network. Finally, the object detection is completed by applying Fully-Connected layers to classify the obtained bounding boxes called Regions of Interest (ROIs). Figure 3 shows a representation of some bounding boxes that contain suspicious areas obtained through the primary diagnosis unit.

#### False positives identification

The performance of the system is evaluated as the average precision (AP) introduced by the Pascal VOC Challenge. The AP is the area under the Precision-Recall curve for detection. It has a constant interval Recall level [0, 0.1, …, 1], and is the mean AP calculated for all classes, as shown in Equations (2, 3).

(2) $$\begin{array}{l} AP=\frac{1}{11}\underset{r\in \left\{0,0.1,\ldots ,1\right\}}{\sum }P_{\mathrm{int}erp}\left(r\right) \end{array}$$

(3) $$\begin{array}{l} P_{\text{int}erp}\left(r\right)=\underset{\tilde{r}:\tilde{r}\geq r}{\max}\;p\left(\tilde{r}\right) \end{array}$$

where, *P*_*interp*_(*r*) is the maximum precision for any recall values greater than *r*, and $p\left(\tilde{r}\right)$ is the measured precision at recall $\tilde{r}$. Then the AP is computed as the average of *P*_*interp*_(*r*) at all recall levels. IoU, defined in Equation 4, is a widely-used metric for evaluating the accuracy of object detectors.

(4) $$\begin{array}{l} IoU\left(A,B\right)=|\frac{A\cap B}{A\cup B}| \end{array}$$

where *A* represents the ground-truth box collected in the Annotation and *B* represents the prediction result of the network. If the estimated *IoU* is higher than the threshold, the predicted results will be considered as positive samples (TP + FP), otherwise as negatives (FN + TN). TP, FP, FN, and TN represent the True Positives, False Positives, False Negatives and True Negatives respectively. Ideally, the number of FPs and FNs should be small and the network must determine how accurately each case can be handled.

Table 1 shows the number of True Positive and False Positive bounding boxes generated by the primary diagnosis unit for each class using the Faster R-CNN detector when the IoU threshold = 0.5. The results evidence a relation of 89.97% TP and 10.03% FP of the total of bounding boxes generated.

**Table 1:** Identification of True positive and false positive bounding boxes generated by the primary diagnosis unit.

**Class**	**True positives**	**False positives**	**Total**
Leaf mold	11022	900	11922
Gray mold	1642	1126	2768
Canker	2226	422	2648
Plague	2246	324	2570
Miner	5198	85	5283
Low temperature	426	51	477
Powdery mildew	314	24	338
Whitefly	380	24	404
Yellow leaf curl	3819	108	3927
Nutritional excess	403	23	426
Total TP	27676 (**89.97%**)^*^		
Total FP		3087 (**10.03%**)^*^	
Total Samples			30763

* *The percentage value corresponds the portion respect to the total*.

**Figure 4:**
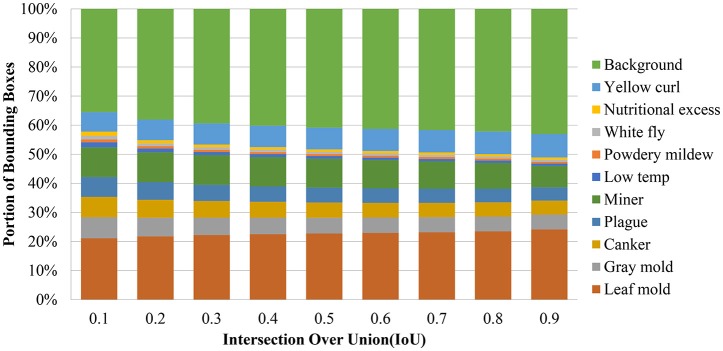
The result of the bounding box detector evidence an unbalance between classes. Each column represents a comparison of the number of bounding boxes of each class using different intersection over union thresholds from 10 to 90%.

The IoU is a parameter that is used to determine whether a detected bounding box is a TP, TN, FP, or FN. However, the number of false positives may vary for each class, due to in part to the complexity and number of samples available. Additionally, they represent a problem mainly caused by the inter- and intra-class variations presented in the dataset. To determine this relationship, we extract the bounding boxes from the primary diagnosis unit and evaluate the detection results with different IoU thresholds. As shown in Figure 4, we notice an unbalance between the positive classes (diseases and pest) and the background class (negative class) is highly visible. In fact, since the number of examples for some classes such as leaf mold and yellow curl virus is relatively high compared to other classes. Consequently, the system tends to give higher priority to cases with a greater source of information.

Notably, it is common that the number of positive samples detected by the primary diagnosis unit decreases as the IoU threshold is increased. However, the impact on the recall should be also considered in terms of the number of false negatives. Since our training data have a large unbalance between classes, we investigate whether changing the IoU threshold value produced any change in the number of positive samples. Figure 4 shows the portion of bounding boxes detected by the primary diagnosis unit with respect to IoUs and classes. The unbalance between the classes from the original training data is not reduced but it becomes even larger as the IoU threshold increases. When one of the target classes contains a much smaller amount of training data than the other target classes, it may be dominated by the others. Especially, if the class has a relatively large intra-class variation and small inter-class variation, its performance will be further degraded. In that case, the detector will produce more false positives for that class and the other classes as well.

**Figure 5:**
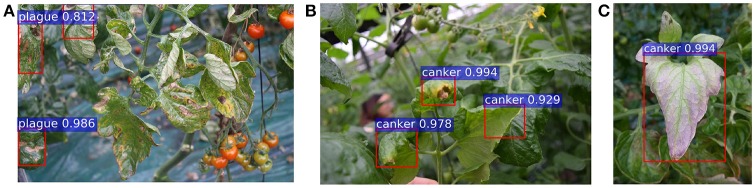
A representation of some false positives generated in the primary diagnosis unit: **(A)** canker samples are detected as plague; **(B)** gray mold samples are detected as canker; **(C)** a low temperature sample is detected as canker.

Figure 5 shows some examples of false positives generated by the primary diagnosis unit. We present cases of canker, gray mold, and low temperature samples that have been misclassified as plague, canker, and canker, respectively. To improve the performance of the entire system, we need to investigate a strategy that allows the system to keep the true positives while handling the false positives.

**Figure 6:**
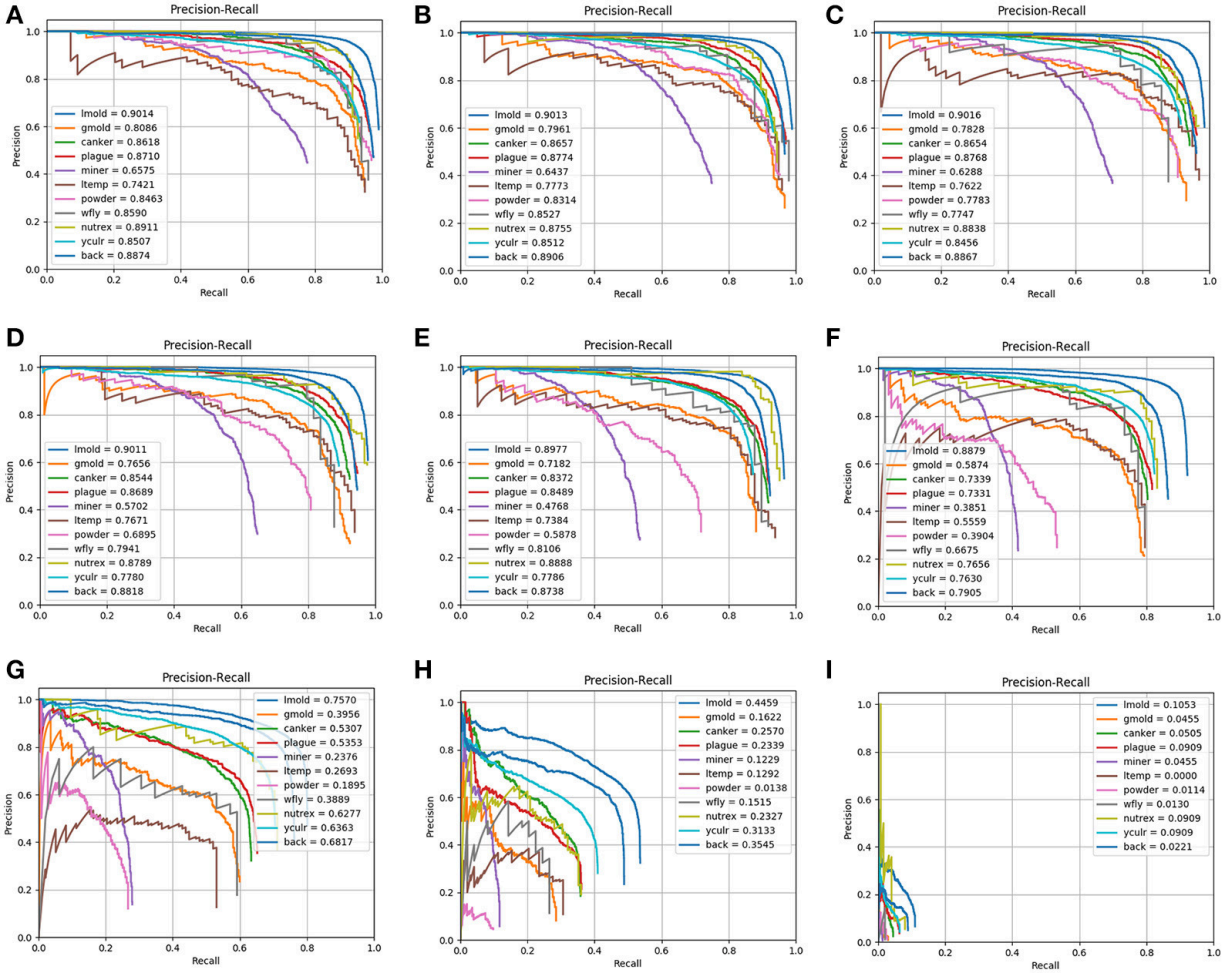
Precision-Recall curves of the primary diagnosis unit (bounding box generation) for different IoU threshold values: **(A)** 0.1; **(B)** 0.2; **(C)** 0.3; **(D)** 0.4; **(E)** 0.5; **(F)** 0.6; **(G)** 0.7; **(H)** 0.8; **(I)** 0.9. Note that the performance decreases as the IoU value is increased.

As can be seen in Figure 6, due to the limited data available, the unbalance between classes results in lower performance. Each representation in Figure 6 shows the precision-recall curves of the primary diagnosis unit using different IoU threshold values from 0.1 to 0.9. The precision-recall curves of the primary diagnosis unit illustrate that classes with more samples tend to be more stable and, therefore they may obtain a higher score. In addition, as the IoU value is increased, the performance of the system decreases and, consequently, some classes tend to be more affected since they may get confused among themselves or with others. This could be the case when more than one pathology is found in the sample area of the plant or is a consequence of various infection status with different visible patterns. Furthermore, we might also argue that there should be a tradeoff between the precision and recall when choosing a proper threshold value for the evaluation.

**Figure 7:**
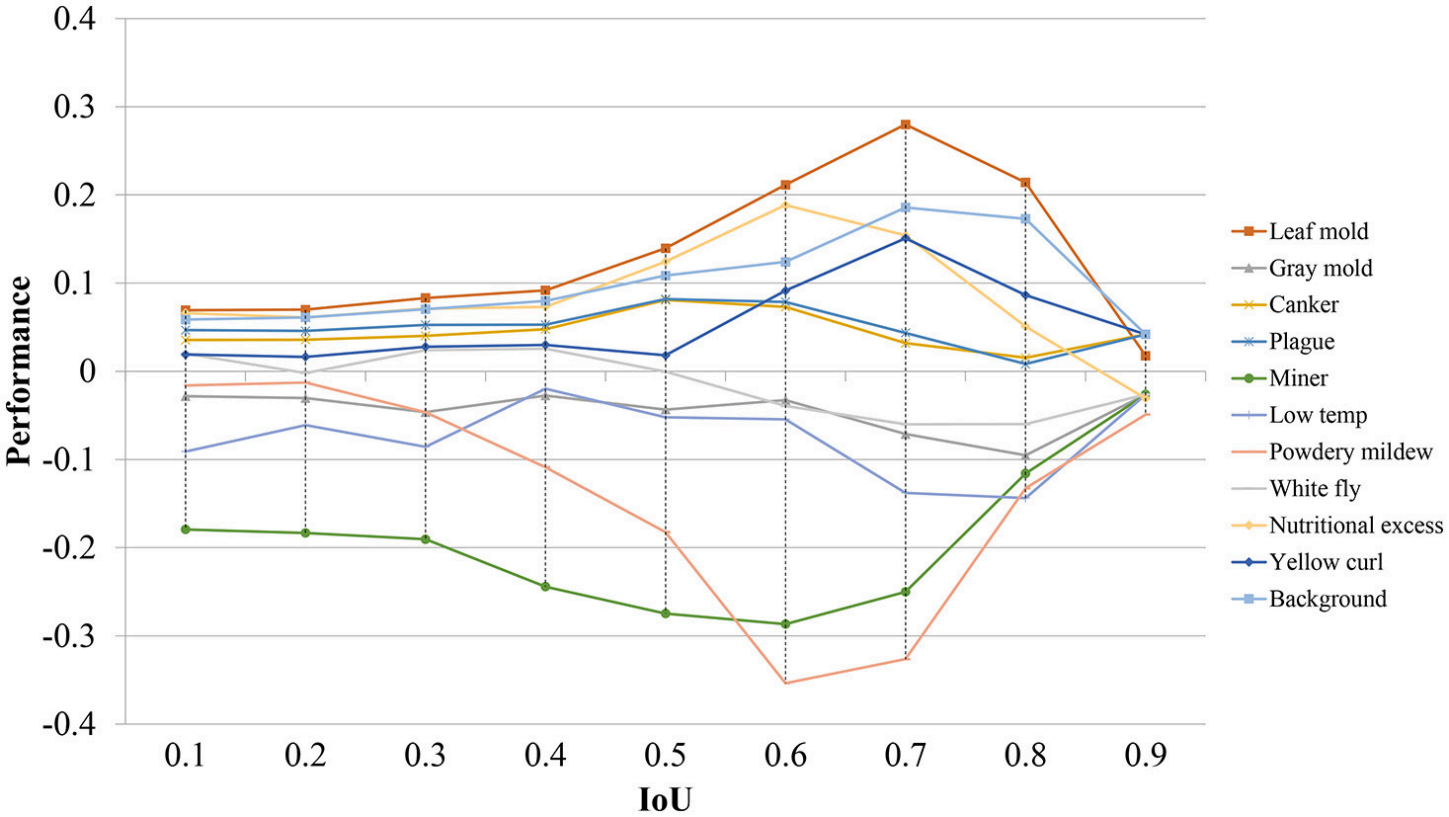
Performance differences of all detected classes in terms of their average precision using different IoU threshold values. Note that some classes experience a positive performance, while others show a negative value.

To visualize the individual performance of each class, we evaluate the average precision at different IoU threshold values. Figure 7 shows that some classes such as leaf mold, canker, plague, yellow curl virus, nutritional excess show even better performance than the mean average precision. However, some critical classes like powdery mildew and miner experience worse performance as the IoU value is increased. These classes represent the challenging pathologies that may cause several detection inconveniences in the primary diagnosis unit.

In order to address the problem of false positives and improve the detector stability and performance of the system, we introduce the secondary diagnosis unit. To achieve that purpose, this unit firstly sets the recall value $\text{R}=\;\frac{\text{T}\text{P}}{\text{T}\text{P}+\text{F}\text{N}}$ and aims to improve the precision value $\text{P}=\;\frac{\text{T}\text{P}}{\text{T}\text{P}+\text{F}\text{P}}$ using the filter bank. (The details of the filter bank are described in the next section).

### Secondary diagnosis unit

**Figure 8:**
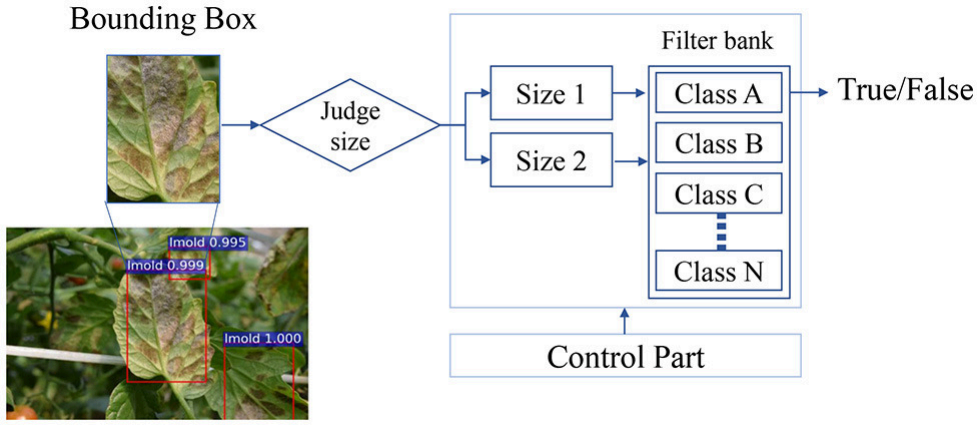
A representation of a CNN filter bank for one class. The input images of the filter bank are the bounding boxes generated in the primary diagnosis unit. A judge step establishes the size of the image prior to its entrance to the CNN. The result is a decision of whether a target belongs to the category as it was detected (True) or not (False).

The generated bounding boxes are very diverse in size and may contain different pathologies. Thus, the set of boxes are extracted and each one adjusted to an appropriate size before training the CNN filter bank. Within the classification block, there is a size adaptation that processes bounding boxes of various sizes and a control block that transfers data to the filter bank based on the information of the previously detected classes. Figure 8 illustrates a general overview of the CNN filter bank.

#### Input data

**Figure 9:**
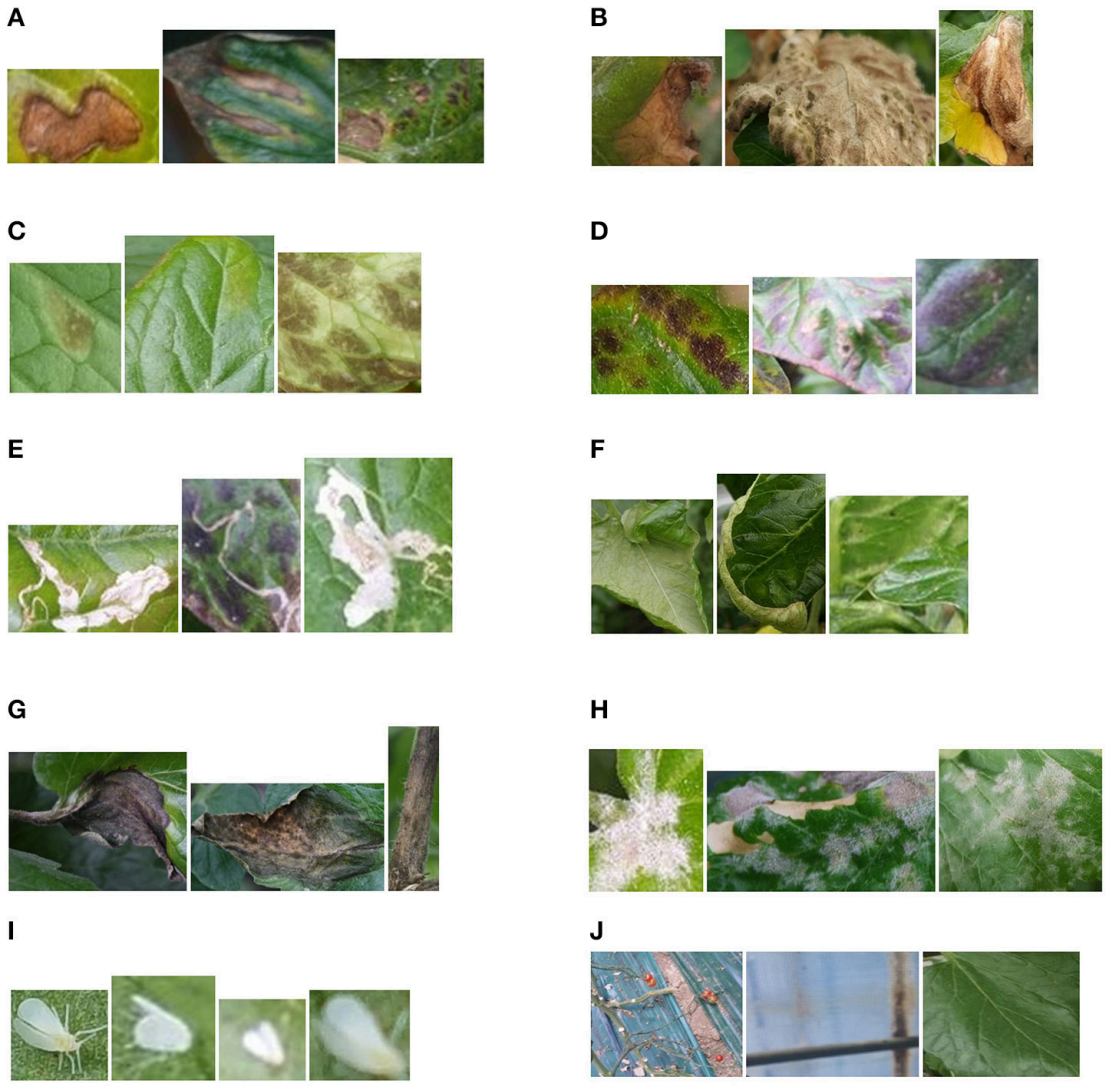
Areas containing suspicious infections due to diseases and pests that are generated by the primary diagnosis unit and used as input to the CNN Filter Banks. **(A)** Canker, **(B)** Gray mold, **(C)** Leaf mold, **(D)** Low temperature, **(E)** Miner, **(F)** Nutritional excess, **(G)** Plague, **(H)** Powdery mildew, **(I)** Whitefly, **(J)** Background.

In this stage, regions that contain bounding boxes generated in the primary diagnosis unit by the Faster R-CNN are firstly extracted from the original images and then consecutively used by the CNN filter bank. They are divided into 10 different types of diseases and pests. Additionally, we include an extra class called “background.” This class basically contains healthy areas of the plant or parts of the main scenario. Figure 9 shows some examples of the bounding boxes used as input of the filter banks.

The number of classification blocks depends on the number of classes to be diagnosed. In addition, another function of the control block is to perform a process of adapting the size of the bounding boxes, before entering their respective CNN classifier. Each CNN determines either True or False values by estimating the probability of a disease or pests that appear in the input image.

#### Filter bank architecture

To address the problem of false positives caused by misclassification, we propose to use the secondary diagnosis unit that includes a CNN filter bank for each category. The added classifier plays a role of a judge that decides whether a bounding box is likely containing the correct target or not. In the CNN filter bank, each CNN directs a specific proposal to a particular object category, which in fact, also includes false positives as negative samples to make the system more robust against intra- and inter-class variations. The characteristics of the filter bank are introduced below.

a) Scale Adaptation

We construct a filter bank which contains *k-CNNs*, where *k* is the number of classes. Every CNN is an independent network but with the same number of parameters. Given a set of bounding boxes for each category, the control block first adapts the sizes of the bounding boxes to two scales: small and large, and feeds them into their respective CNN. To facilitate the process, each box is sampled to 300 × 300 and 500 × 500.

b) Filter Bank

**Figure 10:**
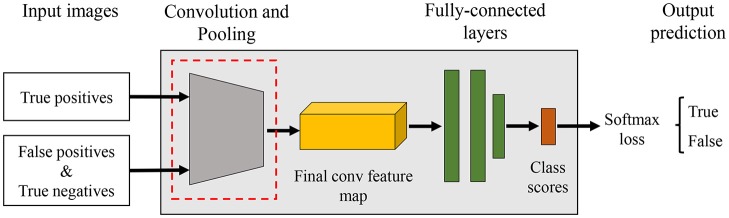
Example of a CNN architecture used in the filter bank. The goal of each CNN is to verify if an input bounding box is likely containing the target category or not, as well as, to make the system more robust against intra- and inter-class variations.

Our *k*-CNNs are implemented in Caffe. For each network, we use a simple CNN architecture with 5 convolutional layers and 3 fully-connected layers. Figure 10 illustrates a representation of a CNN architecture used in the Filter Bank.

To deal with the problem of false positives caused by misclassification, we consider our filter bank-based approach as an additional classifier for each object category. We find it important to train each CNN independently using the detection output (bounding boxes) of a specific category, so the detection should have a higher score on that category. To that effect, each CNN uses bounding boxes specific to one category, which allows to capture intra-class variation.

During the training process, first, the primary diagnosis unit (bounding box generator) is trained on the training dataset. Then, the bounding boxes (set of true positive, false positive and true negative boxes) obtained from the primary diagnosis unit are used to train the secondary diagnosis unit (filter banks). Further, the set of boxes containing the true targets are selected as positives samples and, the false positives along with true negative samples (hard negatives) are used as negative samples. The proposed approach works like a filter whose goal is to preserve bounding boxes with higher recognition rate while eliminating the false positives and negatives from the list. As shown in Figure 10, a CNN structure for class diagnosis is examined, and the final result is a precision value of a specific class performed by a single CNN network. To make the training process effective, both units are trained and optimized consecutively with shared convolution weights.

During testing, using an input image, the primary diagnosis unit generated a set of bounding boxes that contain the object categories. Then, each detection is again classified by the secondary diagnosis unit. As both units share weights, the image feature maps are computed only once during testing.

The advantage of this structure is that it can respond effectively to diseases or pests that appear in the images. Basically, the system consists of a modular architecture that can be adapted to as many categories as required. It is also possible to include more categories simply by adding a CNN to the filter bank.

#### Improving the precision results

The purpose of this technique is to increase the precision score. This is a technique commonly used for object detection, but has been adapted for our application. Therefore, increasing the precision score is the most important factor in measuring the efficiency of this technology.

**Figure 11:**
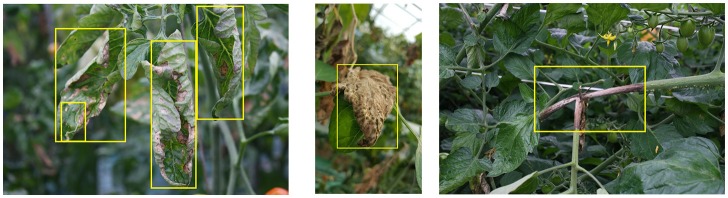
A representation of various images with bounding boxes used for training. The yellow boxes represents the suspicious infected areas of the plant.

Figure 11 shows a representation of images of tomato plants used for learning. The yellow rectangle represents the suspicious areas of the disease or pests located in the foreground, and the rest is considered the background. The areas annotated within the yellow bounding boxes are considered positive samples of their respective class, and the False Positive or True Negative samples are selected either as part of another class and as background of the image. Nevertheless, it is necessary to emphasize that all samples containing the suspicious areas should be annotated, to avoid confusing the system when testing in unseen images.

#### Complexity of the CNN filter bank

**Figure 12:**
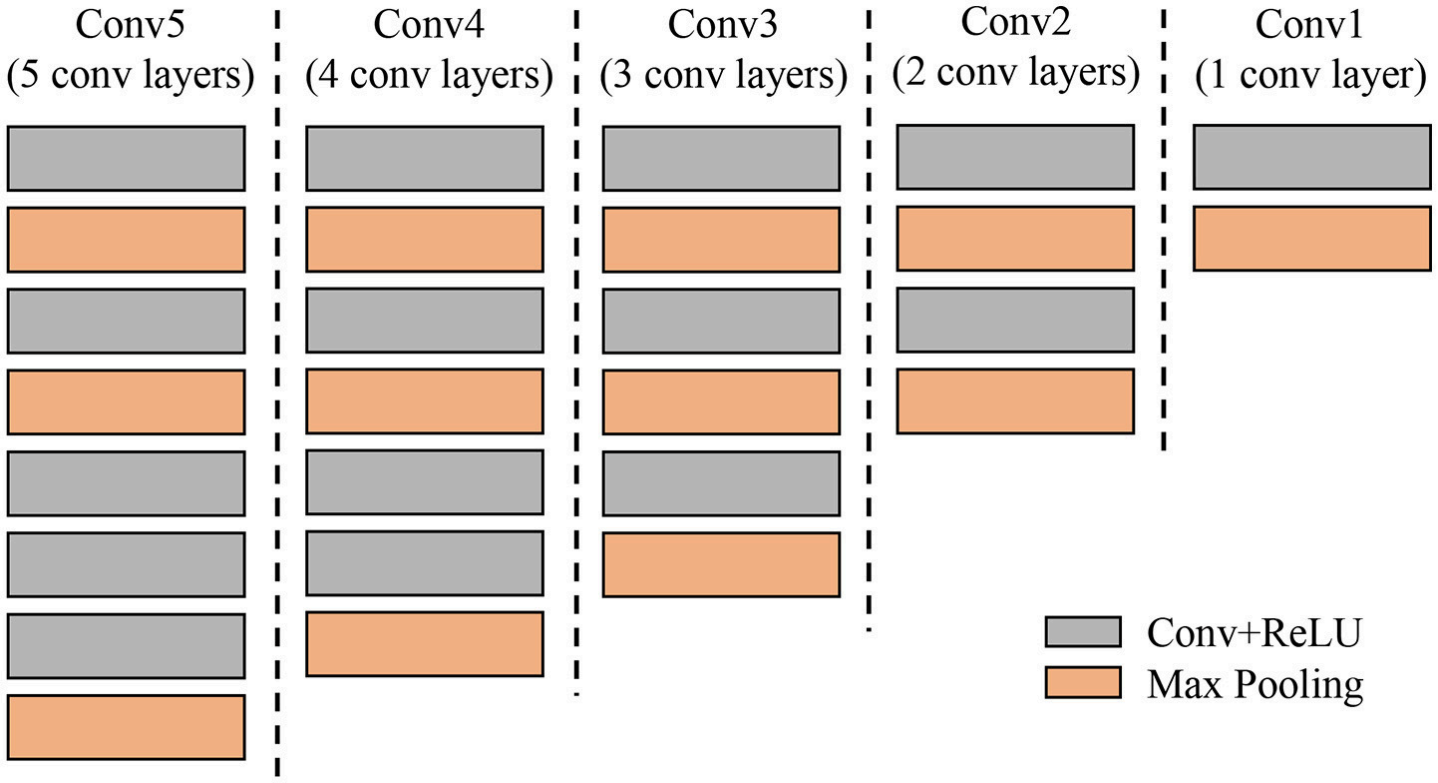
Different designs of Convolutional Neutral Networks used in the experiments to understand the required parameters of the secondary diagnosis unit. Each column represents a single CNN from 5 to 1 convolutional layers (gray color).

The application of a secondary diagnosis unit (CNN Filter Banks) allows the system to achieve higher precision while maintaining a reasonable computational cost. The CNN models of the filter banks are a flexible framework with different design selections. We make several modifications to the architecture to verify the performance of the network. Therefore, we have extended the design to understand the number of layers needed for the system to be accurate enough. Through this technique, we can find a suitable solution for our application without sacrificing system performance. Figure 12 shows different CNN architectures that are further tested in the experimental results. They consist of 5, 4, 3, 2, and 1 convolutional layers respectively. Each convolutional network represents the area within the red bounding box in Figure 12.

### Integration unit

The integration unit (see Figure 1) combines the results from the primary diagnosis unit (bounding box generation) and the secondary diagnosis unit (CNN filter bank). The result of the CNN filter bank is a decision on whether the target corresponds to the category as it was detected (True) or not (False). Next, the integration unit has two main functions: (1) it combines the information of primary and secondary units, and (2) it keeps the True Positive samples and eliminates the False Positives that were misclassified in the first stage. As mentioned earlier, a smaller number of False Positives helps to improve the precision score. The whole process operates autonomously, which allows the system to provide accurate results in real time.

## Experimental results

### Tomato diseases and pests dataset

We conduct experiments on our Tomato Diseases and Pest Dataset, as in (Fuentes et al., 2017b). This dataset consists of approximately 5,000 images collected from several tomato farms located in different areas of South Korea. Diseases and Pest can be developed under different conditions such as climate, location, humidity, etc. Therefore, using simple camera devices, the images were collected in various conditions depending on the time (e.g., illumination), the season (e.g., temperature, humidity), and the place where they were taken (e.g., greenhouse) (Fuentes et al., 2017b). Additionally, our dataset includes images with various resolutions, samples in the early, middle, and last infection status, images containing different infected areas in the plant (e.g., stem, leaves, fruits, etc.), different sizes of plants, objects that surround the plant in the greenhouse, etc. The categories and number of samples for each class are presented in Table 2. The number of annotated samples corresponds to the bounding boxes annotated in the images after applying the following data augmentation techniques: geometric transformations (resizing, crop, rotation, horizontal flipping) and intensity transformations (contrast and brightness enhancement, color, noise). The background class is a transverse class that has been annotated in most of the images, and its bounding boxes are used as negative samples during training the CNN filter bank.

**Table 2:** List of categories included in our tomato diseases and pests dataset and their annotated samples.

**Class**	**Number of Images in the Dataset^a^**	**Number of Annotated Samples (Bounding Boxes)^b^**	**Percentage of Bounding Box Samples (%)**
Leaf mold	1,350	11,922	24.06
Gray mold	335	2,768	5.57
Canker	309	2,648	5.33
Plague	296	2,570	5.17
Miner	339	5,283	10.63
Low temperature	55	477	0.96
Powdery mildew	40	338	0.68
Whitefly	49	404	0.81
Nutritional excess	50	426	0.85
Yellow leaf curl	3,927	3,927	7.90
Background^c^	2,177	18,899	38.03
Total	8,927	49,662	100

a *Number of images in the dataset*.

b *Number of annotated samples after data augmentation*.

c *Transverse category included in every image*.

In addition to the dataset used in (Fuentes et al., 2017b), we have also included a new class that contains images of the “yellow leaf curl” virus. As mentioned earlier, we have identified that one of the main difficulties that limit the system to obtain higher precision is the unbalance between classes due to the conditions and limited data available. This can be evidenced by the number of images that belong to each class, as shown in Table 2 and Figure 4.

### Experimental setup

Our proposed system has been trained and tested on two NVidia GeForce TitanXP GPUs. We conducted experiments on our Tomato Diseases and Pest dataset, using an extensive data augmentation to avoid overfitting. The data has been distributed as follows:

- Primary diagnosis unit: from the whole number of images in the dataset, 80% are used for training, 10% for test and the remaining 10% for validation.
- Secondary diagnosis unit: depending on the number of True Positives and False Positives mentioned in Table 1, we divide them into 80% for training and 20% for test. However, since the number of images for some classes is limited, we include samples from other classes as negative samples in each CNN to avoid problems of class unbalance during training and test.

### Complexity of the CNN filter bank

**Figure 13:**
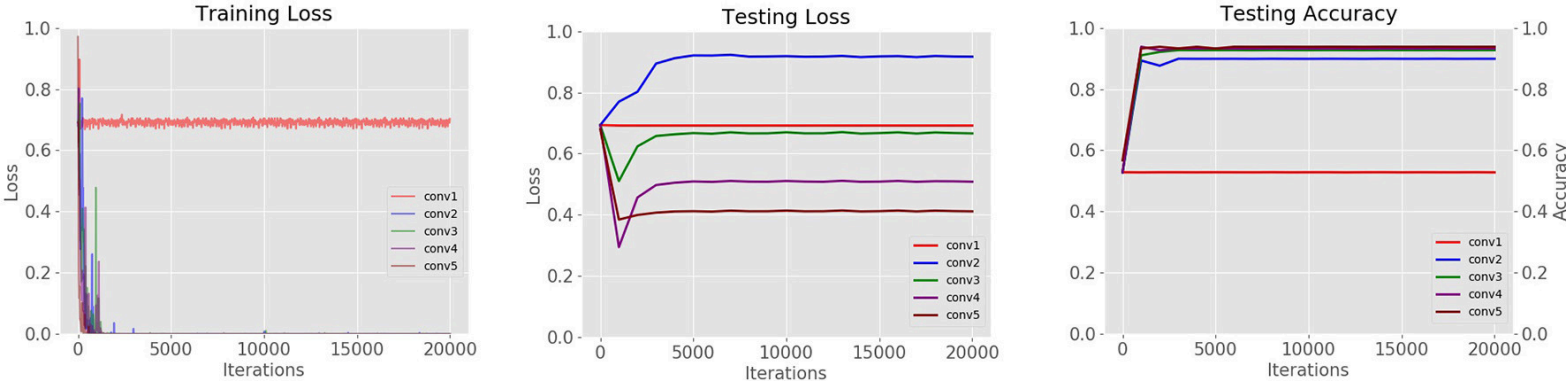
Loss curves of CNN architectures with different layers studied in the refinement filter bank of our proposed approach.

We are interested in observing how the performance changes in different levels of the Convolutional Neural Network. For this purpose, we have trained a set of CNNs with a various number of layers in the filter bank. We found that models with fewer layers are more likely to be overfitted. Since the amount of data is still small for some classes, we also found that although CNNs with one and two layers learn during training, they are not able to generalize well during testing. CNNs with three or four layers show acceptable performance, but a CNN with 5 layers tends to be more stable during testing. The results can be seen in Figure 13.

### Data distribution

**Figure 14:**
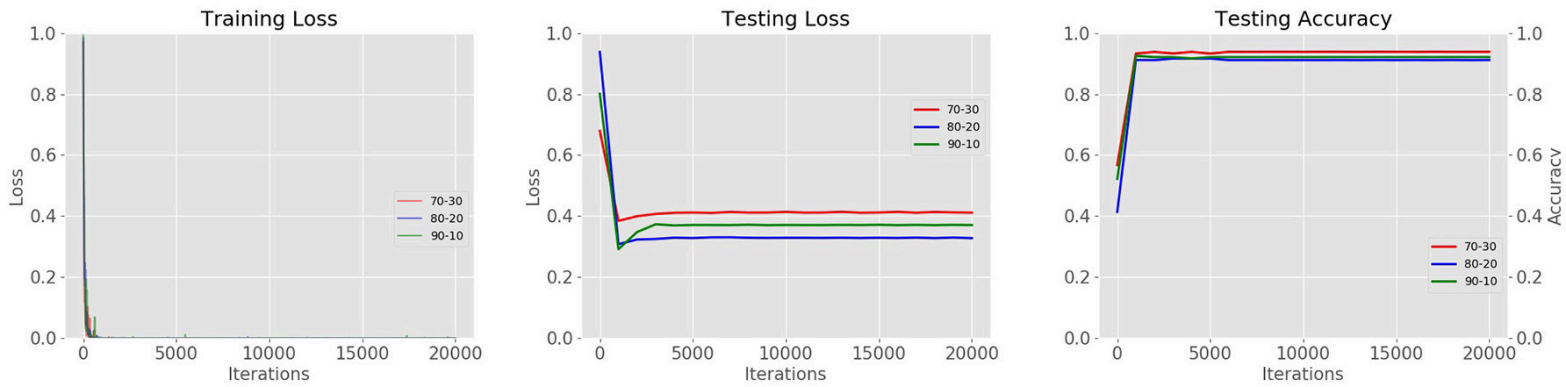
Results of data distribution for cross-validation. Using three different combinations of data (70–30%, 80–20%, 90–10% trainval and testing respectively).

Using different combinations of training-validation (trainval) and testing data, we are also able to find the best combination that allows the system to generate better results. The goal is not only to improve the precision value, but also to propose a system that is stable during training and testing. Therefore, we trained and tested the system with different combinations of data. We found that a distribution of 80% training and 20% testing shows more stability throughout the iterations in the testing loss curve, in contrast to the results of the testing accuracy curve where combination a 70% training, 30% testing shows better performance. The results of data distribution with different combinations are illustrated in Figure 14.

### Quantitative results

Table 3 shows the final results of our refinement system. The comparative values evidence a satisfactory improvement in all classes with respect to our previous results (Fuentes et al., 2017b). The mean Average Precision demonstrates an improvement of about 13%. This is, in fact, due to the implementation of the secondary diagnosis unit (CNN filter bank) that allows the system to filter misclassified samples focusing mainly on the reduction of false positives.

**Table 3:** Comparative results of our proposed approach with the previous system (Fuentes et al., 2017b).

**Class**	**FRCNN (VGG-16)**	**Refinement Filter Bank (Proposed)**	**Difference of accuracy**
Leaf mold	0.9060	0.9205	0.0145
Gray mold	0.7968	0.8910	0.0942
Canker	0.8569	0.9376	0.0807
Plague	0.8762	0.9710	0.0948
Miner	0.8046	0.9947	0.1901
Low temperature	0.7824	0.9821	0.1997
Powdery mildew	0.6556	0.9963	0.3407
Whitefly	0.8301	0.9929	0.1628
Nutritional excess	0.8971	0.9893	0.0922
Yellow leaf curl	0.8500	0.9500	0.1000
Mean AP	0.8255	0.9625	0.1370

The number of samples and variations are another key points that influence in the final results. For example, in the case of gray mold, the number of samples is smaller than leaf mold. Moreover, the gray mold class shows a high intra-class variability that could confuse the system with other classes (See Figure 4).

### Does the CNN filter bank help?

The input images of the filter bank are the set of bounding boxes generated by the Bounding Box Generator. The control part sets the size of the images before entering the CNN filter bank. The result is defined as “True” if the image falls into the same category as it was detected or “False” otherwise.

**Figure 15:**
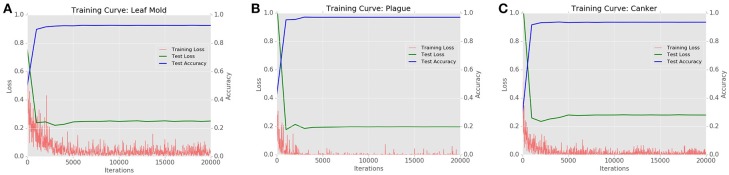
Training curves generated by the CNN filter bank for the most challenging classes of the system **(A)** Leaf mold with AP: 92%, **(B)** Plague with AP: 97%, **(C)** Canker with AP: 93%.

Figure 15 shows the Training Loss, Testing Loss, and Testing Accuracy of the CNN filter bank for the most challenging classes in the dataset such as leaf mold, plague, and canker.

Due to the implementation of a secondary diagnosis unit, the results have been substantially improved compared to the previous results reported in (Fuentes et al., 2017b). Therefore, we might argue the importance of the CNN filter bank toward reducing the number of false positives. As presented in Table 3, the mean Average Precision has been increased in approximately 13% compared to the best results generated by the Faster R-CNN with a VGG-16 feature extractor in (Fuentes et al., 2017b).

An additional benefit of using a second diagnosis unit is the easy configuration of the framework. The CNN Filter Bank that is composed by a set of CNN architecture, as the one shown in Figure 10. This modular architecture is able to add another network if the study requires including more classes by changing the structure shown in Figure 8.

## Conclusion and future works

In this work, we have proposed a framework based on deep neural networks that performs onto promising object-specific bounding boxes for efficient real time recognition of diseases and pests of tomato plants. Our detector uses images captured in the field by various camera devices and process them in real time. The detector is composed of three units: A primary diagnosis unit (bounding box generator) first learns to propose bounding boxes with size, location, and class through a Region-based Neural Network trained with the input images. The promising bounding boxes that belong to each class are then used as input to the secondary diagnosis unit (CNN filter bank) for verification. This secondary unit filters misclassified samples by training independent CNN classifiers for each class. The result of the CNN Filter Bank is a decision on whether the target corresponds to the category as it was detected (True) or not (False) otherwise. Finally, an integration unit combines the information from the primary and secondary units by keeping True Positive samples and eliminating False Positives that were wrongly misclassified in the first stage. By this implementation, the proposed approach outperforms our previous results by a margin of 13% mean Average Precision in the task of tomato diseases and pest recognition. Furthermore, our system is able to deal with the problems of false positives generated by the Bounding Box Generator, and class unbalances that appear especially in datasets with limited data. We expect that our work will significantly contribute to the agricultural research area. Future works will focus on extending our approach to other types of crops.

## Author contributions

AF designed the study, performed the experiments, and data analysis, and wrote the paper. DP and SY advised on the design of the system and analyzed to find the best method for efficient recognition of diseases and pests of tomato plants. JL provided the facilities for data collection and contributed with the information for the data annotation.

### Conflict of interest statement

The authors declare that the research was conducted in the absence of any commercial or financial relationships that could be construed as a potential conflict of interest.
